# Mediterranean Diet Pyramid: A Proposal for Italian People

**DOI:** 10.3390/nu6104302

**Published:** 2014-10-16

**Authors:** Annunziata D’Alessandro, Giovanni De Pergola

**Affiliations:** 1 Endocrinologist, General Practitioner. General Medicine ASL BA/4 D.S.S. 8, viale Japigia 38/G, Bari 70126, Italy; 2 Department of Biomedical Sciences and Human Oncology, Section of Internal Medicine and Oncology, University of Bari “Aldo Moro”, School of Medicine, Policlinico, Piazza Giulio Cesare 11, Bari 70124, Italy; E-Mail: gdepergola@libero.it

**Keywords:** Mediterranean diet pyramid adult, sourdough bread, glycemic index, glycemic load

## Abstract

Bread was a staple in the traditional Mediterranean diet of the early 1960s, as well as nowadays; however, it was a stone ground sourdough bread in Nicotera and probably in the Greek cohorts of the Seven Countries Study. In the present review, the nutritional characteristics of this food are analyzed in relation to its protective effects on coronary heart disease, metabolic diseases and cancer. According to our traditions, cultural heritage and scientific evidence, we propose that only cereal foods with low glycemic index (GI) and rich in fiber have to be placed at the base of the Mediterranean diet pyramid, whereas refined grains and high GI starchy foods have to be sited at the top.

## 1. Introduction

In 1992, the United States Department of Agriculture reported nutritional recommendations for the American population in the form of pyramid [1], helping people to make good dietary choices with the aim of reducing the risk of chronic diseases. The growing scientific evidence that the Mediterranean diet protects against coronary heart disease, some kinds of cancers and other chronic diseases and that it guarantees longevity in adults led some scientists to represent this dietary pattern as a pyramid. The success of this geometric figure in the nutritional field is possibly due to its ability to give information about the frequency and the proportion of food intake and at the same time representing a healthy diet. Over the years, some Mediterranean diet pyramids were elaborated by the USA [2,3] and by Mediterranean countries, such as Greece [4], Spain [5] and Italy [6], to give their populations dietary advice based on both cultural heritage and scientific evidence in order to prevent chronic diseases and promote longevity. The importance of the Mediterranean diet for a healthy lifestyle has been mainly recognized in the scientific world, whereas we believe this topic should be addressed by politicians. 

**Figure 1:**
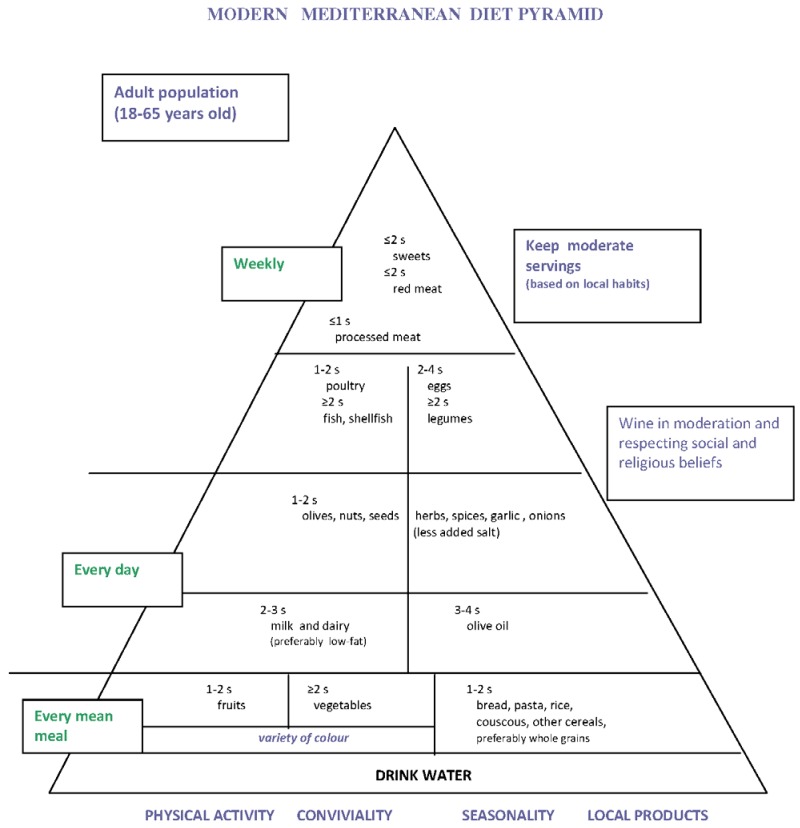
Modern Italian diet pyramid presented during the third CIISCAM conference in Parma, Italy, on November 3, 2009 [6]. (CIISCAM: Centro Interuniversitario Internazionale di Studi sulle Culture Alimentari Mediterranee.)

However, here, we more specifically analyze the Modern Italian Mediterranean diet pyramid presented during the third conference of CIISCAM (Centro Interuniversitario Internazionale di Studi sulle Culture Alimentari Mediterranee) held in Parma, Italy [6] (Figure 1), as a consensus position of some scientist experts in nutrition. This pyramid contributed substantially to building a model of a unified Mediterranean diet pyramid for the Mediterranean area [7]. The aim of this review is to suggest an improvement of that pyramid with some changes concerning, above all, cereal food that may be important for the health of Italian people.

## 2. Cereal Foods in the Traditional Mediterranean Diet of the Early 1960s 

Fruit, vegetables and cereals are the basis of the Mediterranean diet. As regards cereals, one or two servings per meal as bread or pasta or rice or couscous, preferably as whole grains, are suggested [6] (Figure 1). Undoubtedly, the traditional Mediterranean diet of the early 1960s was rich in cereals. In the Crete cohort of the Seven Countries Study, which had the lowest mortality rate for coronary heart disease [8], the average amount of bread consumption was 380 g per person and per day [9]. A similar amount was reported for the Corfu cohort (450 g person^−1^ day^−1^) [9]. Ancel Keys commented that, in both areas, bread was mostly dark, and, in Crete, it was often made from whole wheat and barley [10]. In general, the dietary pattern of the Crete and Corfu cohorts was very similar because of the high intake of olive oil, vegetables, fruit, cereals, the moderate intake of dairy foods, fish, eggs, wine at meals and the low meat intake. A dietary survey conducted in a rural population of a Southern Italian town, Nicotera, showed that their diet in 1960 was quite similar to that found in the two rural areas of Crete and Corfu in Greece [11]. The average intake of cereals, in 40–59-year-old men, was 455 g person^−1^ day^−1^; their bread, generally brown bread, was made of wheat flour, stone ground in the 18 mills of the town, and was consumed at every meal [12]. Therefore, the diet of the middle-aged men sampled to be enrolled in the Crete, Corfu and Nicotera cohorts of the Seven Countries Study in the early 1960s had a high percentage of total daily energy intake from bread (Crete in 1960: mean 28.9% ± 7.1%; Corfu in 1961: mean 38.1% ± 7.0% [10]; Nicotera in 1960: median 32.1% [11]).

In Nicotera, bread was leavened with a sourdough starter [13], probably a Type I traditional sourdough, because the addition of baker’s yeast for leavening, required for Type II and Type III sourdoughs [14,15], was not indicated. It is possible that bread was leavened with sourdough also in Crete and Corfu in the early 1960s [16]. Simopoulos refers that it was a feature of the traditional Greek diet until the end of the 1950s [17]. However, it should be noted note that the westernization of dietetic habits, which started at the beginning of the 1960s, was slower in the poor rural areas than in the richer areas of the country [18] and in the lower social classes than in the upper ones [19].

Spontaneous sourdough is a dough of flour and water that, left for several hours at room temperature, allows the growth of a composite ecosystem of lactic acid bacteria and yeasts [20]. The backslopping, namely the continuous daily refreshments with the addition of new flour and water, keeps the microorganisms in an active state [15]. Yeasts are the major producers of CO_2_ in sourdough [20], so they are primarily responsible for leavening [15]. The lactic acid bacteria, both homo-fermentative and hetero-fermentative species, are responsible for dough acidification, the former producing lactic acid, the latter lactic acid plus CO2, acetic acid and/or ethanol [15]. The final pH is about 4.0, and the fermentation quotient (lactic/acetic acid ratio) is from 3.3 to 5.6 in 18 out of 19 traditional/typical Italian breads [21]. The sourdough fermentation of wheat flour lowers the GI of bread, reducing starch digestibility, mostly through the formation of organic acids [22,23]. The mechanism responsible for the slow absorption of starch in the presence of lactic acid is the inhibition of amylolytic enzymes [24] or a reduction of starch bioavailability, because of the interaction between starch and gluten [25], whereas acetic acid delays the gastric emptying rate [26]. The whole wheat bread average GI is 71, and the glycemic load (GL) for a 30-g serving size is nine [27]; the white wheat flour bread GI is 71, and the GL of a 30-g serving size is 10 [27]; while the GI of sourdough wheat bread is 54, and its GL for a 30-g serving size is eight [27]. If we accept the suggestion of the Harvard Medical School that ranks food GIs ≤55 as low, between 56 and 69 as moderate and ≥70 as high GI foods, therefore, sourdough wheat bread would be considered a low GI food [28]. Whole wheat bread and white wheat bread give the same postprandial response of glucose and insulin in type 2 diabetic patients [29,30]; however, the inclusion of a high percentage of intact or partially milled cereal kernels in the flour reduces the glycemic response of bread [31,32,33]. These data have a possible biological and technical explanation: wheat germ contains a natural amylase inhibitor that is destroyed by the passage through the roller mill. Standard wholemeal flour (not stone ground meal flour) is a reconstituted flour after passage through the roller mill. Therefore, wholemeal flour is hydrolyzed at a rate identical to white flour [34]. 

Scazzina evaluated the metabolic effects of four breads prepared from two different wheat flours (whole or white) through two different leavening techniques (sourdough and *Saccharomyces cerevisiae*). Both sourdough fermented breads gave glycemic responses significantly lower than the corresponding breads leavened with *Saccharomyces cerevisiae* in eight healthy volunteers. The presence of fiber did not influence the glycemic potential of breads [35]. In 16 glucose-intolerant subjects, who had randomly received either a meal containing bread (70% durum wheat semolina and 30% corn flour) leavened with sourdough or a meal containing bread leavened with baker’s yeast, sourdough bread induced a significantly lower plasma glucose response at 30 min and a significantly smaller incremental area under the curve at 0–30 and 0–60 min in comparison to bread leavened with baker’s yeast. Accordingly, the plasma insulin response of sourdough bread showed significantly lower values at 30 min and a significantly smaller incremental area under the curve at 0–30 min [36]. The sourdough wholemeal wheat breads resulted in the lowest postprandial glucose and insulin response among four tested breads (white wheat bread, wholemeal wheat bread, sourdough wholemeal bread and wholemeal bread made with xylanase) [37].

Sourdough fermentation was more efficient than yeast fermentation in reducing phytate content in whole wheat bread (−62% and −38%, respectively) [38], because the reduction of the pH value provided favorable conditions for the endogenous cereal phytase activity [39]. Phytate, which is widely represented in whole grains and legumes, but also in oil seeds and nuts [40], strongly bound to metal cations of Ca, Fe, K, Mg, Mn and Zn, making them insoluble and, thus, unavailable as nutritional factors [41]. Furthermore, Katina *et al*. commented: “With sourdough processes, the mouthfeel and the palatability of wholemeal bread can be improved without removing any nutritionally important components” [42].

## 3. Traditional Mediterranean Diet of the Early 1960s as a Low GI and GL Diet 

Since many fruits and vegetables, beans, pasta, dairy foods and nuts have a low GI [27,43], the Mediterranean traditional diet with sourdough wholemeal bread can be qualified as a low GI diet. This type of diet can also be a good guide to get a low GL diet, if the intake of starchy foods is controlled. 

Low GI/GL diets offer many health advantages in comparison with high GI/GL diets on body weight, blood lipid levels, risk of type 2 diabetes mellitus (T2DM), coronary heart disease and cancer. 

As regards the effects of high GI diets on body weight, this type of diet raises glucose and insulin in the early postprandial period (0–2 h) more than low GI diets, and the excess of insulin lowers blood glucose in the postprandial period (3–5 h), thus leading to excessive hunger. The decrease of plasma glucose and the increased hunger have been associated with increased activity in specific brain regions related to food intake, reward and craving in overweight or obese men. The assessment was done by using arterial spin-labeling functional magnetic resonance imaging [44]. In 14 healthy subjects, an activation of limbic-striatal brain regions with a concomitant increasing desire for high-calorie foods was evident when a mild hypoglycemia occurred during hyperinsulinemic euglycemic-hypoglycemic clamp [45]. A recent meta-analysis of clinical trials evaluated the relationship between glycemic response and markers of health; when food intake was limited controlled or *ad libitum*, low GL diets were significantly associated with lower body weight under free living conditions if a GL reduction by ≥17 g glucose equivalent/day occurred and most consistently when the GL reduction was by >42 g glucose equivalent/day [46]. However, despite these data, observational studies show conflicting results about the relationship between GI/GL and body mass index [47,48,49,50].

High GI/GL diets have an unfavorable effect on serum lipid, influencing cardiovascular disease. In cross-sectional analyses of two large cohort studies [51,52], a large trial [53] and a sample of the Nurses’ Health Study [54], GL was inversely associated with HDL-cholesterol and directly associated with triglycerides. In 5830 non-diabetic subjects aged 20–70 of the Health Worker Cohort Study, the adjusted odds ratios in the highest *versus* the lowest quartile of dietary GL were 1.78 for low HDL-cholesterol (<40 mg/dL for men; <50 mg/dL for women) (*p* for a trend of 0.002) and 1.85 for high triglycerides (≥150 mg/dL) (*p* for a trend of 0.01) [52]. In two studies [52,53], high dietary GI was positively associated with both lower HDL-cholesterol and higher triglyceride concentrations. In a small cross-sectional study in which dietary GI/GL were calculated accurately from three-day dietary records, the highest concentration of HDL-cholesterol and the lowest of triglycerides and insulin were observed in the lowest GI tertile (*p* < 0.01) [55]. An increase in insulin resistance, which, in turn, causes an increase in triglycerides and a decrease in HDL-cholesterol, can explain the effects of high GI/GL diets on blood lipids. In the early postprandial period (0–2 h after the meal), the rapid absorption of carbohydrates after a high GI meal leads to a relatively high blood glucose level and a high insulin/glucagon ratio. In the late postprandial period (4–6 h after the meal), the counter-regulatory hormones restore normal glycemia and cause a marked increase in free fatty acid concentration [56]. Elevated glucose, insulin and free fatty acids induce insulin resistance [56].

High GL diets have been associated with an increased risk of developing T2DM in several large prospective studies. A recent systematic review and meta-analyses of 24 prospective cohort studies with 7.5 million person-years of follow-up showed that dietary GL ranged from ~60 to ~280 g per daily intake of 2000 kcal (8.4 MJ) and was positively associated with a T2DM relative risk of 1.45 (95% CI: 1.31, 1.61) for a 100-g increment in dietary GL (*p* < 0.001) [57]. The potential mechanisms whereby high GL diets, over a period of years, could increase the risk of T2DM include an increase in insulin demand following hyperglycemia that, in turn, leads to a loss of pancreatic function (due to β-cell exhaustion or toxicity of hyperglycemia) and an increased insulin resistance induced by free fatty acids, produced in the late postprandial period by counterregulatory hormones. The progressively higher glucose levels induced by a high GL depend on the degree of underlying insulin resistance being more evident in obese, inactive or genetically susceptible people [58].

Several prospective studies have evaluated the relationship between dietary GI/GL and the risk of coronary heart disease. Four systematic reviews and meta-analyses [59,60,61,62], including an Italian prospective study [63], showed that high dietary GL significantly increases the risk of coronary heart disease in women, but not in men. A greater decrease in HDL-cholesterol and a greater increase in triglycerides in women than in men in response to a high GI/GL diet [64], due to sex-related differences in lipid metabolism, can explain these results. Furthermore, the hazards ratio for coronary heart disease is higher in diabetic women than in men [65]. Interestingly, in the EPICOR (long-tErm follow-up of antithrombotic management Patterns In acute CORonary syndrome patients) study, increasing carbohydrate intake from high GI foods was significantly associated with greater risk of coronary heart disease in women, whereas increasing carbohydrate intake from low GI foods was not [63]. The dietary GI can influence the risk of coronary heart disease through inflammation and oxidative stress. Low GI diets are associated with lower C-reactive protein [53] and lipid peroxidation markers [66] in comparison to high GI diets in cross-sectional studies. 

Several case-control and prospective epidemiological studies investigated the relationship between GI/GL diets and cancer development. Chronic hyperinsulinemia could be the link between long-term consumption of high GI/GL diets and cancer [67]. Chronic hyperinsulinemia may promote cancer through abnormal stimulation of multiple cellular signaling cascades and increasing the bioactivity of insulin-like growth factor 1. Insulin, by reducing sex hormone binding globulin levels, increases the estrogens bioavailability that, in turn, can promote cellular proliferation and inhibit apoptosis in breast epithelium and endometrium [68]. Moreover, hyperinsulinemia increases proinflammatory cytokines and oxidative stress that, in turn, can promote malignancy and neoplastic progression [68]. Systematic reviews and meta-analysis showed conflicting results regarding the involved cancer sites [69,70,71,72,73]. However, a large nationwide population-based case-control study showed that GI was positively associated with the risk of prostate cancer, and a high GL significantly increased the risk of colorectal and pancreatic cancers [74].

Another peculiarity of wholemeal sourdough bread should be considered, that is its richness in fiber. High intake of cereal fiber reduces the risk of coronary heart disease [75], T2DM [76], obesity [77] and colorectal cancer [78]. Cereal fiber reduces inflammatory markers [79,80] and improves insulin sensitivity [81]. Whole grain cereals, but not refined grains, reduce the risk of coronary heart disease, gastrointestinal cancer [82] and T2DM [82,83]. The protective mechanisms of whole grain cereals against the risk of developing chronic diseases depend on bioactive compounds and dietary fiber, mainly contained in the bran and germ fractions, that are lost in the milling process [84]. Interestingly, fiber and oligosaccharides of whole grain wheat have also a prebiotic effect on gut microbiota [85,86] involved in systemic low-grade inflammation and progression of chronic metabolic diseases [87]. 

## 4. Proposal of Mediterranean Diet Pyramid for Italian People 

**Figure 2:**
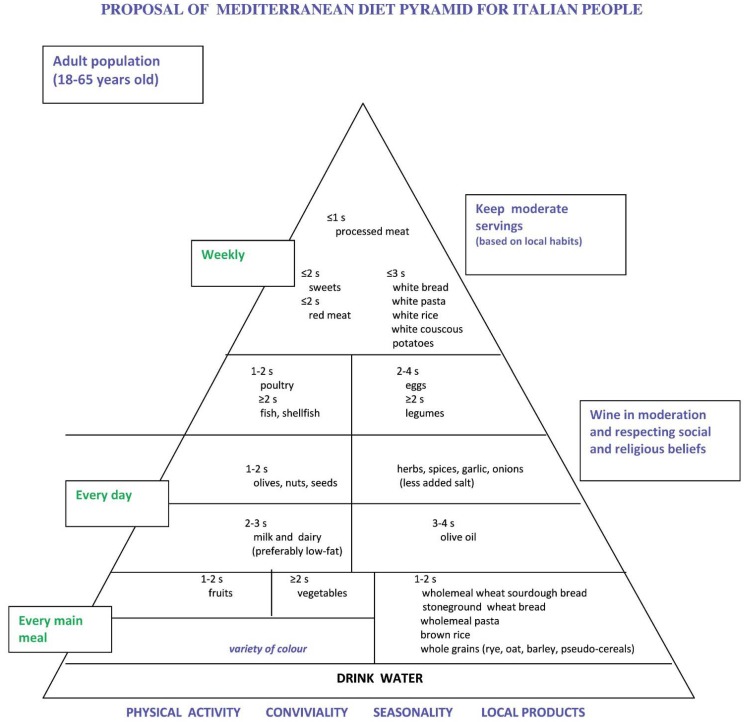
Proposal of the Mediterranean diet pyramid for Italian people.

As a result of the above considerations, it can be said that at the base of the Mediterranean diet pyramid there should be low GI, fiber-rich cereals, such as wholemeal wheat sourdough bread, stone ground wheat bread, wholemeal pasta, brown rice and whole kernel cereal grains. At the top of the pyramid, there should be cereal foods with high GI and that are poor in fiber, such as white bread, white rice, white couscous and potatoes and even a food with low GI, but that is poor in fiber, such as white pasta (Figure 2). Wholemeal sourdough pizza (with low-fat cheese) and wholemeal couscous could be placed also at the base of the pyramid, if a low GI for these meals is demonstrated in the future. In this way, we would get a better choice in the cereal type than in the one induced by the word “preferably” of the present Mediterranean diet pyramid. 

## 5. Conclusions 

In conclusion, we state that the Italian Mediterranean diet pyramid presented during the third CIISCAM conference in Parma [6] (Figure 1) should be modified as far as the position of cereal foods is concerned, because they are a heterogeneous category if we consider their effects on health, as has been shown above. The proposed new pyramid (Figure 2) can help Italian people avoid the risk of many common chronic diseases, as it is based not only on our cultural heritage and traditions, but also on scientific evidence. There are many problems for the implementation of the proposed pyramid, because of the low availability of the suggested types of cereal foods. However, we have a long tradition in making sourdough bread; there are more than 200 different types of traditional bread manufactured throughout Italy, and almost one-third of them are leavened with sourdough [88]. Bread is a staple in Mediterranean countries [89,90], and in the Italian diet, it accounts for almost 40% of the total cereal intake [91]. A nutrition policy that acts by increasing the availability of wholemeal (possibly from stone ground flour) sourdough bread through the activity of artisan bakeries is essential and needs to be supported by politicians. The intake of whole grain cereal foods rich in fiber should be promoted, and many efforts should be made to promote healthy Mediterranean foods, thus avoiding the “westernization” process of food habits [92] that increases risk factors of chronic diseases. The adoption of the traditional Mediterranean diet could impede the increase of and even reduce many of these risk factors, above all, the main cardiovascular risk factors [16]. Even a little reduction of these risk factors by adopting the traditional Mediterranean diet at a population level can reduce cardiovascular disease more than pharmacological interventions in people at high risk [93]. Furthermore, the traditional Mediterranean diet is a sustainable diet, as it has a low environmental impact and is healthy for consumers, while the current Mediterranean dietary patterns are not sustainable [94]. Is our past diet the future of our health? 
